# 3′UTR shortening of profibrotic genes and reversibility of fibrosis in patients with end‐stage right ventricular failure

**DOI:** 10.1002/ctm2.1017

**Published:** 2022-09-09

**Authors:** Rahul Neupane, Katarzyna A. Cieslik, Keith Youker, Suresh Selvaraj Palaniyandi, Ashrith Guha, Rajarajan A. Thandavarayan

**Affiliations:** ^1^ DeBakey Heart and Vascular Center Houston Methodist Hospital Houston Texas; ^2^ Division of Cardiovascular Sciences, Department of Medicine Baylor College of Medicine Houston Texas; ^3^ Division of Hypertension and Vascular Research, Department of Internal Medicine, Henry Ford Health System Detroit Michigan; ^4^ Department of Physiology, Wayne State University Detroit Michigan

**Keywords:** alternative polyadenylation, fibrosis, right ventricle failure, 4HNE

1

Dear Editor,

Cardiac fibrosis, a prominent feature of pressure overloaded right ventricle (RV) in pulmonary hypertension (PH), is fundamental to the development of right ventricle failure (RVF). The molecular mechanisms driving fibrosis is not well understood especially at the level of RNA regulation. We found that a defect in alternative polyadenylation (APA) of profibrotic genes is a driving factor that induces fibrosis. Oxidative stress‐mediated reactive aldehydes, such as 4‐hydroxy‐2‐nonenal (4HNE) contribute to fibrosis and extracellular matrix (ECM) remodelling. However, their role in APA is unknown. For the first time, our study identified the missing link between 4HNE‐mediated stress and the regulation of 3′UTR by APA in the progression of cardiac fibrosis using end‐stage RVF specimens.

We established a pronounced fibrosis state in RVF compared to the controls (Figure S1A). We assessed the relative distal Poly‐A site (dPAS) usage in these samples (Figure S1B). COL1A, FN1 and TGFβR1 showed significantly lesser usage of dPAS and hence, a shortening in their 3′UTR length in RVF (Figure S1C). Due to 3′UTR shortening in profibrotic genes, they lose microRNA (miRNA) or long non‐coding RNA (lncRNA) regulatory sites, thus promoting RNA stability, protein expression, and as a consequence, ECM accumulation.^1^ Finally, we validated the increased protein expression of these 3′UTR shortened genes, COL1A and FN1, in overall RVF tissues and Vimentin‐positive fibroblasts (Figure S1D–G).

Previous studies have shown 4HNE as a contributor to various pathologies such as LV hypertrophy, PH‐induced RV fibrosis, remodelling, myocardial infarction‐induced lipid peroxidation, dilated cardiomyopathy and cardiotoxicity.^2^ We showed a substantial decrease in the expression and activity of aldehyde dehydrogenase 2 (ALDH2), an enzyme metabolizing 4HNE, (Figure S2A–C) and a significant accumulation of 4HNE adducts (Figure S2D and E) in cardiomyocytes and fibroblasts in RVF. Stress was shown to cause global 3′UTR shortening in proliferating and differentiating cells.^3^ Here, our study provides the first evidence that establishes strong association between 4HNE‐mediated stress and its contribution to fibrosis through 3′UTR shortening in profibrotic genes.

Fibrosis is mainly enforced by activating cardiac fibroblasts into proliferative α‐SMA‐positive myofibroblasts, which cause excessive collagen secretion in the ECM. We characterized the fibroblasts isolated from RVF and showed their profibrotic nature (Figure 1). We showed significant shortening in 3′UTR of major ECM genes, such as COL1A and FN1. Additionally, 3′UTRs of TGF‐β1 and its receptor TGFβR1 and NFκB, a transcription factor regulating proinflammatory and profibrotic responses which is also implicated in oxidative stress, were also shortened in RVF (Figure 1B). The shortening in these major profibrotic genes correlated with their higher protein expression in RVF fibroblasts (Figure 1C). Further, they were marked by α‐SMA, showed accumulation of COL3A and had higher contractility (Figure 1C–F).

**FIGURE 1:**
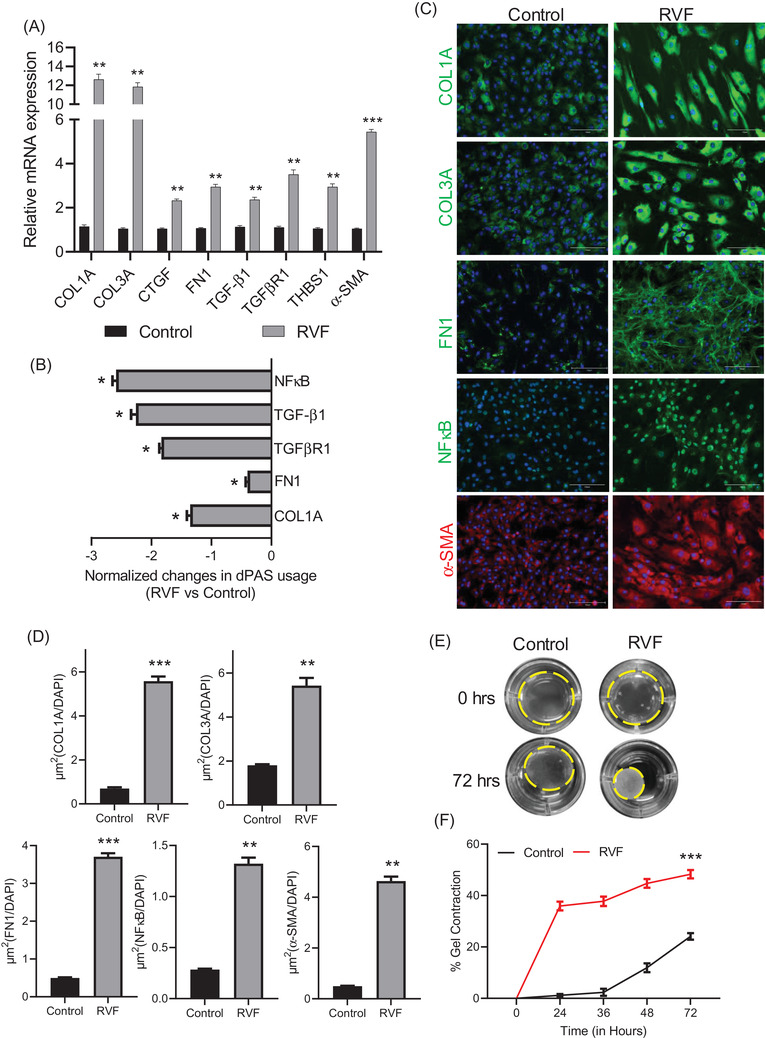
APA changes in profibrotic genes in fibroblasts from RVF patients. (A) Bar plot showing the mRNA expression of major profibrotic genes COL3A, COL1A, CTGF, TGF‐β1, FN1, TGFβR1, THBS1, and α‐SMA in RVF and control fibroblasts by RT‐qPCR. (B) Plot showing 3′UTR changes in COL1A, FN1, TGFβR1, TGF‐β1, and NFκB genes in isolated fibroblasts using RT‐qPCR. (C) Immunofluorescence staining using anti‐COL1A, COL3A, FN1, NFκB, and α‐SMA antibodies in RVF and control fibroblasts. Scale bar, 150 μm. (D) Quantification of fluorescent area stained by antibodies in Figure (C). (E) Representative photographs of collagen gel contraction by RVF and control fibroblasts after 0 and 72 h. (F) Percentage gel contraction by fibroblasts with time. Student's *t*‐test was used to analyse the data. Data are presented as mean ± SEM; *n* = 3, *p < .05 and **p < .01

We demonstrated ALDH2 deficiency and its reduced catalytic activity (Figure 2A–C) leading to 4HNE adduction (Figure 2D and E) and increased intracellular superoxide accumulation (Figure 2F–H) in these profibrotic RVF fibroblasts. Lipid peroxidation and 4HNE accumulation are well‐established in fibrosis progression.^4^ Previous studies show a positive correlation between 4HNE adducts, fibrosis and TGF‐β1 expression. Most importantly, it has been directly shown to induce fibrosis by regulating TGF‐β1 through its regulator‐activator protein‐1 (AP‐1) and activating NFκB signalling.^5^ Here, we showed a possible role of 4HNE in 3′UTR regulation of profibrotic genes, including TGF‐β1, its receptor TGFβR1, and NFκB in the stressed RVF fibroblasts. Stress was shown to induce global 3′UTR shortening,^3^ so 4HNE‐mediated oxidative stress may induce 3′UTR shortening in profibrotic genes by regulating core APA machinery proteins. Previously, we and others have reported 3′UTR regulation of profibrotic genes as a result of dysregulation in APA machinery proteins in human diseases like heart failure^1^ and chronic obstructive pulmonary disease (COPD)^6^ where oxidative stress is prevalent.^2^, ^4^

**FIGURE 2:**
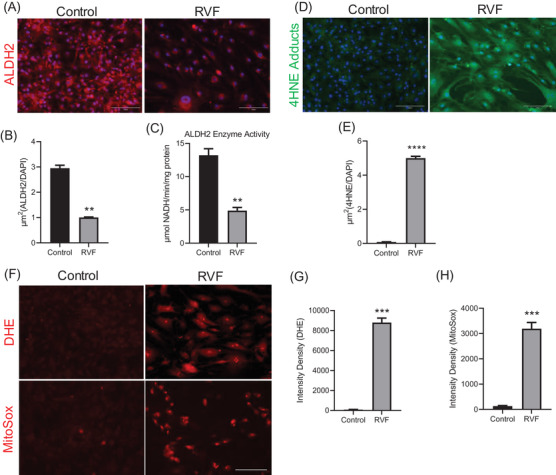
ALDH2 expression and activity in isolated RVF fibroblasts. (A) Immunofluorescence staining using ALDH2 antibody in RVF and control fibroblasts. Scale bar, 150 μm. (B) Plot displaying quantification of ALDH2 expression in the fibroblasts. (C) ALDH2 enzyme activity in proteins from the fibroblasts. (D) Immunofluorescence staining using anti‐4HNE adducts antibody in RVF and control fibroblasts. Scale bar, 150 μm. (E) Plot displaying quantification of 4HNE adducts expression in the fibroblasts. (F) Detection of superoxide by fluorescence in red channel by using Dihydroethidium or MitoSox™ Red in RVF and control fibroblasts. Scale bar, 150 μm. (G, H) Quantification of the fluorescent intensity density signal in Figure (F). Student's *t*‐test was used to analyse the data. Data are presented as mean ± SEM; *n* = 3, *p < .05 and **p < .01

ALDH2 is well known to metabolize and prevent 4HNE accumulation and ROS release, ultimately alleviating oxidative stress, inflammation and fibrosis. Alda‐1 is an agonist of ALDH2 which has been shown to increase its enzymatic activity. ALDH2 activation by Alda‐1 was shown to restore cardiac function and lessen fibrosis in the in vitro model^7^ and also in the myocardial infarction model of heart failure.^8^ Moreover, Gomes and colleagues also reported that counteracting 4HNE‐mediated fibroblast proliferation by Alda‐1 treatment^8^ lessens ventricular remodelling. Likewise, we showed downregulation of ECM genes COL1A, COL3A, and FN1 and NFκB after Alda‐1 treatment in RVF fibroblasts (Figure 3A, C and D). Alda‐1 treatment also downregulated the expression of α‐SMA and TGF‐β1 while the expression of its receptor TGFβR1 did not change. An important finding here was the 3′UTR lengthening of profibrotic genes, including TGFβR1, after ALDH2 activation by Alda‐1, which provided direct evidence of APA regulation by 4‐HNE (Figure 3B). The 3′UTR lengthening and downregulation of profibrotic genes correlated with the decrease in contractility of these RVF fibroblasts after Alda‐1 treatment (Figure 3E and F). mRNA isoforms with longer 3′UTR are negatively regulated by miRNAs, lncRNAs and RBPs to restrict protein expression.^9^ Previous studies have shown selective degradation of isoforms with longer 3′UTR during recovery.^3^ However, the stress‐induced shorter 3′UTR isoforms escape degradation and sustain their abundance post‐stress.^10^ Hence, addressing APA and ensuring 3′UTR lengthening in shortened profibrotic genes during treatment and recovery of cardiovascular diseases, including RVF, becomes critical to ensure the reversibility of fibrosis.

**FIGURE 3:**
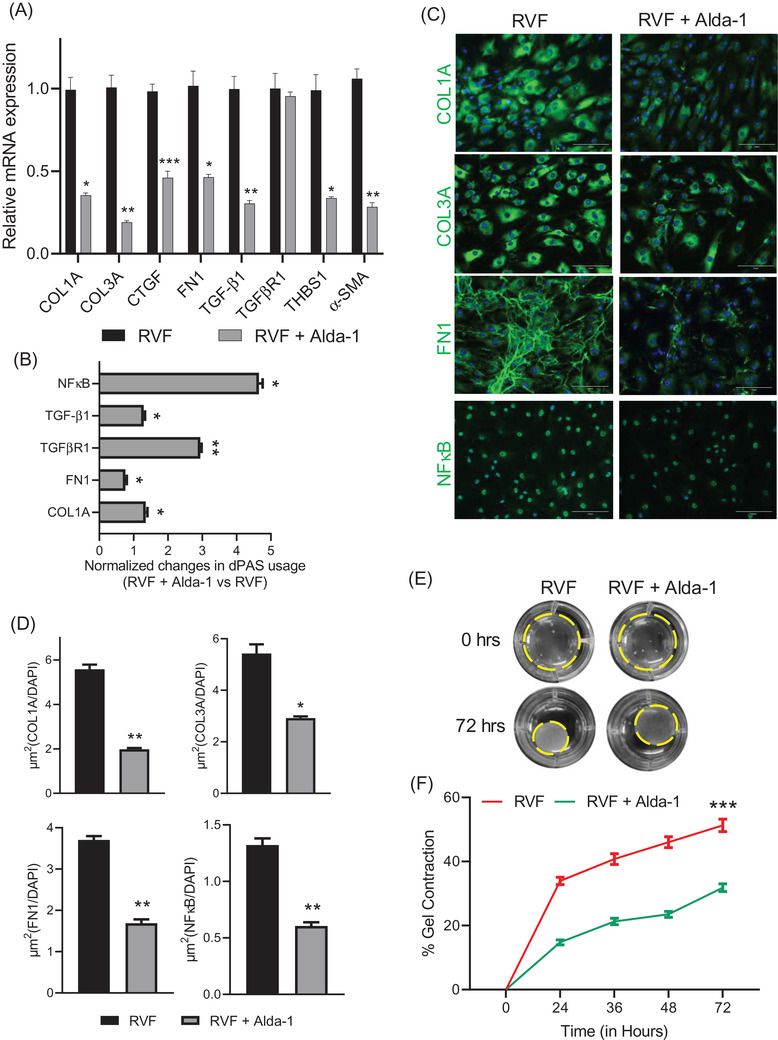
3′UTR changes in profibrotic genes in isolated RVF fibroblasts after treatment with Alda‐1. (A) mRNA expression of COL3A, COL1A, CTGF, TGF‐β1, FN1, TGFβR1, THBS1, and α‐SMA in RVF fibroblasts after Alda‐1 treatment relative to untreated by RT‐qPCR. (B) 3′UTR changes in COL1A, FN1, TGFβR1, TGF‐β1, and NFκB genes in RVF fibroblasts treated with Alda‐1 compared to the untreated using RT‐qPCR. (C) Immunofluorescence staining using anti‐COL3A, COL1A, FN1, and NFκB antibodies in RVF fibroblasts with or without Alda‐1 treatment. Scale bar, 150 μm. (D) Quantification of fluorescent area stained by antibodies in Figure (C). (E) Representative photographs of collagen gel contraction by RVF fibroblasts after 0 and 72 h of Alda‐1 treatment. (F) Percentage gel contraction by RVF fibroblasts after Alda‐1 treatment with time. Student's *t*‐test was used to analyse the data. Data are presented as mean ± SEM; *n* = 3, *p < .05 and **p < .01

We also observed increased ALDH2 expression and enzymatic activity in RVF fibroblasts after Alda‐1 treatment (Figure 4A–C). In addition, a sharp decrease in 4HNE adduct formation (Figure 4D and E) and accumulated levels of superoxide (Figure 4F–H) in the treated RVF fibroblasts were seen. This confirmed the ALDH2‐mediated clearing of reactive aldehydes 4HNE produced due to ongoing oxidative stress. Thus, the ALDH2 activation directly lessened oxidative stress, counteracted 4HNE adducts and reduced the tissue remodelling capabilities of the fibroblasts through 3′UTR changes in profibrotic genes. However, further studies are needed to layout the concrete mechanism of how 4HNE or oxidative stress regulates APA in progressing fibrosis during RVF.

**FIGURE 4:**
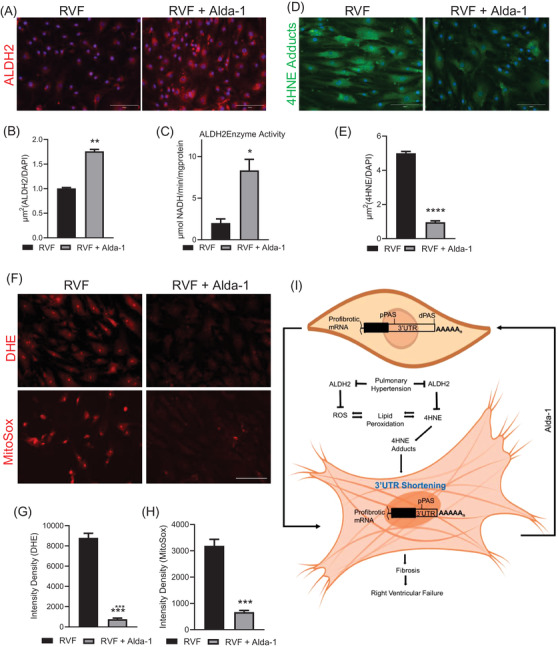
ALDH2 expression and activity in isolated RVF fibroblasts after treatment with Alda‐1. (A) Immunofluorescence staining using ALDH2 antibody in RVF fibroblasts treated with or without Alda‐1 for 48 h. Scale bar, 150 μm. (B) Plot showing the quantification of ALDH2 expression in the fibroblasts. (C) ALDH2 enzyme activity using proteins from treated and untreated RVF fibroblasts. (D) Immunofluorescence staining using anti‐4HNE adducts antibody in RVF fibroblasts treated with or without Alda‐1. Scale bar, 150 μm. (E) Plot showing the quantification of 4HNE adducts expression in the fibroblasts. (F) Detection of superoxide by fluorescence in red channel by using Dihydroethidium or MitoSox™ Red in Alda‐1‐treated and ‐untreated RVF fibroblasts. Scale bar, 150 μm. (G, H) Quantification of the fluorescent intensity density signal in Figure (F). Student's *t*‐test was used to analyse the data. Data are presented as mean ± SEM; *n* = 3, *p < .05 and **p < .01. (I) The model illustrating the regulation of APA by 4HNE to the progression of cardiac fibrosis.

In summary, we demonstrated that ALDH2 activation alone was sufficient to alleviate fibrosis in vitro, suggesting its potential to be used as a treatment strategy to target the fibrotic process to improve RV function in PH‐induced RVF. In addition, considering oxidative stress and its effect on the 3′UTR shortening, new tools may be developed to monitor the progression of RV failure. Developing these tools may lead to therapeutics to prevent the onset of RV failure and improve the quality of life for patients with PH.

## CONFLICT OF INTERESTS

There are no conflicts of interest.

## Supporting information

TABLE S1 Primers used for RT‐qPCR
FIGURE S1 Gene expression and 3′UTR changes in profibrotic genes in RVF
FIGURE S2 ALDH2 expression and activity in RVF

Supplementary material
