# Supplementary material for: 3′UTR shortening of profibrotic genes and reversibility of fibrosis in patients with end‐stage right ventricular failure
Source: Clin Transl Med. 2022 Sep 9;12(9):e1017. doi: 10.1002/ctm2.1017 (PMC9460478; doi:10.1002/ctm2.1017)
Supplement: Supplementary file 1 — TABLE S1 Primers used for RT‐qPCR FIGURE S1 Gene expression and 3′UTR changes in profibrotic genes in RVF FIGURE S2 ALDH2 expression and activity in RVF [file CTM2-12-e1017-s002.docx]

**3’UTR shortening of profibrotic genes and reversibility of fibrosis in patients with end-stage right ventricular failure**

Rahul Neupane^1^, Katarzyna A Cieslik^2^, Keith Youker^1^, Suresh Selvaraj Palaniyandi^3,4^, Ashrith Guha^1*^, Rajarajan A Thandavarayan^1*^

^1^ DeBakey Heart and Vascular Center, Houston Methodist Hospital, Houston, TX, USA.

^2^Division of Cardiovascular Sciences Department of Medicine, Baylor College of Medicine, Houston, TX 77030, USA.

^3^Division of Hypertension and Vascular Research Department of Internal Medicine Henry Ford Health System Detroit MI.

^4^Department of Physiology Wayne State University Detroit MI.

*Running Title: 3’UTR shortening of profibrotic genes in RVF*

*Corresponding author:

Dr. Rajarajan A. Thandavarayan, M. Pharm, Ph.D,

Assistant Research Professor of Cardiology,

Houston Methodist Hospital, Houston, Texas, USA 77030.

Email: [ramirthalingamthandavarayan@houstonmethodist.org](mailto:ramirthalingamthandavarayan@houstonmethodist.org)

Dr. Ashrith Guha, M. D,

Assistant Professor,

Houston Methodist Hospital, Houston, Texas, USA 77030.

Email: [gashrith@houstonmethodist.org](mailto:gashrith@houstonmethodist.org)

**Materials and methods:**

**Human RV tissues collection**

Human tissue samples were collected from the right ventricular wall of the patients undergoing double heart and lung transplantation (RVF samples). In addition, control RV tissue samples were collected from the human donors that were not used for the transplantation because of non-cardiovascular reasons. The fresh tissue samples were collected, dissected into small pieces, and immediately flash-frozen in liquid nitrogen and transferred to -80°C for long-term storage. Some tissue samples were also fixed in 2% paraformaldehyde, dehydrated using increasing concentrations of alcohol, cleared the alcohol in xylene, and finally infiltrated by paraffin for the embedding. This institutional protocol was approved by the Houston Methodist Hospital Institutional Review Board [IRB (2)0511-0100].

**Immunofluorescence staining and quantification**

Paraffin-embedded RV tissue sections of control and RVF patients were deparaffinized and rehydrated following standard protocol. Heat mediated antigen retrieval was performed in sodium citrate buffer (pH 6.0, Sigma-Aldrich, USA). The retrieved slides were blocked using blocking buffer (ChemCruz Biotechnology, USA) at room temperature for 1 to 2 hours. Then, the slides were stained with antibodies against Vimentin, ALDH2 (Santa Cruz Biotechnology, USA), 4HNE adducts (Calbiochem, USA), COL1A, and FN1 (Proteintech, USA). For cultured cells, control and RV failure-derived fibroblasts were grown to 80% confluency and fixed with 4% formaldehyde (Sigma-Aldrich, USA). The cells were stained with anti-ALDH2, -4HNE adducts, -COL1A, FN1, -collagen type III alpha (COL3A) (Proteintech, USA), -NFKB (Santa Cruz Biotechnology, USA), and -α-SMA (Sigma-Aldrich, USA) antibodies. Images were acquired using Olympus AX70 microscope (Olympus, Tokyo, Japan) and EVOS^TM^ M500 microscope (Thermofisher Scientific, USA) at 20X and 40X magnifications. The images were quantified as the area or the intensity density stained by the antibodies using the ImageJ (NIH) software following the NIH guidelines as described previously [1].

**Fibrosis assessment in tissue sections**

The RV tissue sections from control and failure patients were deparaffinized, rehydrated, and stained with Masson's trichrome kit (Sigma-Aldrich, USA) to stain ECM deposition and quantify the degree of fibrosis. Multiple images were taken at a magnification of 10X and 20X to represent the overall tissue sections with an Olympus AX70 microscope (Olympus, Tokyo, Japan). Fibrosis was represented as the total area stained by blue over the red and quantified using the methods described previously [2]. We calculated fibrosis in each sample following this method and established increased fibrosis in RVF (19.35 ± 4.722%, **p* < 0.05, n = 4) compared to the controls (Figure S1A).

**Cardiac fibroblasts isolation, culture, characterization, and treatment with Alda-1**

RV failing hearts were collected in cold phosphate-buffered saline (PBS) and cardiac fibroblasts were isolated from the minced tissues [1]. The cell suspension was seeded in culture dishes in Fibroblast Growth Medium – 3 (Promocell, USA). Human cardiac ventricular fibroblasts (Promocell, USA) were cultured similarly and used for control fibroblasts. On passage 2, the cultured cells were stained with the established markers of fibroblasts – FSP1, Vimentin, COL1A, and COL3A using immunofluorescence staining. More than 95% of the cells stained positively for these markers while staining negatively for the endothelial cell marker, CD31. The RV failure fibroblasts were grown to 80% confluency and serum was starved for 12 hours before the treatment. Then, the fibroblasts were treated with 20 µM of Alda-1 (Sigma-Aldrich, USA) for 48 hours in serum-free medium and collected for RNA isolation or fixed with 4% formaldehyde for the immunofluorescence staining.

**ALDH2 enzyme activity assay**

Protein samples from control and RVF tissues or isolated fibroblasts were extracted using RIPA buffer (Boston BioProducts, USA) supplemented with protease and phosphatase inhibitors (Thermofisher Scientific, USA). ALDH2 enzyme activity was determined by recording the conversion of NAD+ to NADH as described previously [3]. Proteins were quantified by the bicinchoninic acid method (Thermofisher Scientific, USA) and an equal amount of protein (50 µg – 100 µg) was used to determine the enzyme activity. The protein samples from each group were added with 2.5 mM NAD+ (Sigma-Aldrich, USA) and 50mM of sodium pyrophosphate buffer (Sigma-Aldrich, USA) in triplicates. 10mM acetaldehyde (Sigma-Aldrich, USA) was added to initiate the reaction and the absorbance was taken at 340nm to monitor NADH formation from NAD+ using Tecan Spark microplate reader (Tecan, Switzerland). Finally, ALDH2 enzyme activity was presented as nmol NADH/min/mg of protein.

**Staining using superoxide indicators and quantification**

Fibroblasts derived from control and RVF hearts were grown to 80% confluency, and some of them were treated with Alda-1. Live cells were incubated with 5 µM of Dihydroethidium or MitoSox^TM^ Red (Thermofisher Scientific, USA) for 20 - 30 minutes at 37°C. Intracellular superoxide oxidizes the non-fluorescent Dihydroethidium and MitoSox^TM^ to generate oxidized products which can bind nucleic acid and emit fluorescence. The cells were washed with PBS 3 times after the incubation and imaged in the red channel using the EVOS^TM^ M500 microscope (Thermofisher Scientific, USA). The images were quantified as the intensity density stained by the dyes using the ImageJ (NIH) software following the NIH guidelines.

**RNA isolation and RT-qPCR to calculate distal PAS (dPAS) usage**

Tissue samples and isolated cardiac fibroblasts were homogenized in QIAzol Lysis Reagent (Qiagen, USA). Subsequently RNA was extracted using the RNeasy Mini Kit (Qiagen, USA). qScript® cDNA SuperMix (Quantabio, USA) kit was used to construct cDNA and real-time PCR was performed using CFX96^TM^ Real Time System (Bio-Rad, USA). To calculate the dPAS usage, two pairs of primers were designed for each gene targeting an open reading frame and a region proximal to the distal PAS. The primers represented the total transcripts and the mRNAs with longer 3’UTR respectively. The dPAS usage for each group was calculated as ΔCT = CT_distal_ − CT_total_ as described previously [4, 5]. Data were presented as differences respective to control by calculating ΔΔCT= ΔCT_average control_ − ΔCT_average_ _target_. A negative ΔΔCT indicates lesser usage of dPAS and thus 3’UTR shortening while a positive value indicates lengthening compared to the control.

Table 1. Primers used for RT-qPCR

| Gene | Forward Sequence | Reverse Sequence |
| --- | --- | --- |
| Human_COL1A | GTGCGATGACGTGATCTGTGA | CGGTGGTTTCTTGGTCGGT |
| Human_COL1A long | GTGAGGGAGACAGACACCTG | GTGTTCTGGGGATTCAGGAG |
| Human_FN1 | CCGTGGGCAACTCTGT | TGCGGCAGTTGTCACAG |
| Human_FN1 long | TTGCTAGTTTACCGTTCAAGAGT | TTCAATGAAGGAAAGGTGGA |
| Human_TGF-β1 | TACCTGAACCCGTGTTGCTCTC | GTTGCTGAGGTATCGCCAGGAA |
| Human_TGF-β1 long | AAGGTGAGGAAACAAGCCCAG | ACTATCCCCCACTAAAGCAGGT |
| Human_TGFβR1 | ACGGCGTTACAGTGTTTCTG | GCACATACAAACGGCCTATCTC |
| Human_TGFβR1 long | TTTGTGCAGGATTCTTTAGGCTT | GGCTTCTCAGTATCATTCGACTT |
| Human_NFκB | GCAGCACTACTTCTTGACCACC | TCTGCTCCTGAGCATTGACGTC |
| Human_NFκB long | TTCCCCCTTTTCTGCATTTTGC | GAGGTCATCAATTTGCTTTTCCT |
| Human_COL3A | TGGTCTGCAAGGAATGCCTGGA | TCTTTCCCTGGGACACCATCAG |
| Human_CTGF | CTTGCGAAGCTGACCTGGAAGA | CCGTCGGTACATACTCCACAGA |
| Human_α-SMA | CTATGCCTCTGGACGCACAACT | CAGATCCAGACGCATGATGGCA |
| Human_THBS1 | GCTGGAAATGTGGTGCTTGTCC | CTCCATTGTGGTTGAAGCAGGC |

**Collagen gel contraction assay**

Fibroblast derived from control and RV failure hearts (+/- 20µM Alda-1 for 48 hours) were subjected to collagen gel contraction assay to evaluate the contractility. 50,000 cells from each group were resuspended in Dulbecco's modified eagle medium (Thermofisher Scientific, USA) and mixed with rat collagen type I (R&D systems, USA) to achieve a final concentration of 1.5 mg/mL of collagen and a final volume of 0.5 mL. 1 – 5 µL of 1M sodium hydroxide was added to balance the pH. The suspension of cells and collagen was added to a 24-well culture plate and incubated at 37°C for 30 - 60 minutes until gelatinization. A 20 µL pipette tip was used to separate the gel from the edges of the well gently without damaging the gel. Then, the cells were cultured in DMEM supplemented with 10% fetal bovine serum (FBS) and the photographs were taken every 12 – 24 hours for 5 days.

**Statistical Analyses**

Results are presented as mean ± standard error mean (SEM). Unpaired Student's t-test was performed to test statistical differences between two groups. All the statistical analyses were carried out using the Graph Pad Prism software V9.0 (Graph Pad Software Inc., USA).

**Figure Legends:**

**Figure S1. Gene expression and 3’UTR changes in profibrotic genes in RVF. (A)** Representative photomicrographs of control and RVF heart tissue cross-sections stained with Masson's Trichrome. Scale bar, 50 µm. **(B)** An illustration depicting primers used to detect total (P1) and long variants (P2) transcript. **(C)** Plot shows 3’UTR changes in COL1A, FN1, and TGFβR1 genes in RVF hearts compared to the controls by dPAS using RT-qPCR. Immunofluorescence co-staining of COL1A with Vimentin **(D)** or FN1 with Vimentin in RVF **(F)** and control heart tissues. Insets show the magnified area of the section in the lower panel. Arrows show Vimentin positive fibroblast cells. Scale bar, 20 µm. Quantification of COL1A **(E)** or FN1 **(G)** expression in all the cell types (*Left*) and specifically in Vimentin positive fibroblasts (*Right*) in RVF and controls. Data are presented as mean ± SEM. Student's t-test was used to analyze the data; n = 3, * *p* < 0.05 and ** *p* < 0.01.

**Figure S2. ALDH2 expression and activity in RVF. (A)** Immunofluorescence co-staining using ALDH2 and Vimentin antibodies in RVF and control heart tissues. Insets show the magnified area of the section in the lower panel. Arrows show Vimentin positive fibroblast cells. Scale bar, 20 µm. **(B)** Bar plot showing quantification of ALDH2 expression in all the cell types (*Left*) and specifically in Vimentin positive fibroblasts (*Right*). **(C)** ALDH2 enzyme activity using proteins from control and failing RV tissues. **(D)** Immunofluorescence co-staining using anti-4HNE adducts and Vimentin antibodies in RVF and control heart tissues. Insets show the magnified area of the section in the lower panel. Arrows show Vimentin positive fibroblast cells. Scale bar, 20 µm. **(E)** Bar plot showing quantification of 4HNE adducts expression in all the cell types (*Left*) and specifically in Vimentin positive fibroblasts (*Right*). Data are presented as mean ± SEM. Student's t-test was used to analyze the data; n = 3, * *p* < 0.05 and ** *p* < 0.01.

**References:**

1. Neupane, R., et al., Cleavage stimulating factor 64 depletion mitigates cardiac fibrosis through alternative polyadenylation. Biochem Biophys Res Commun, 2022. 597: p. 109-114.

2. Wang, G., et al., Role of Endothelial and Mesenchymal Cell Transitions in Heart Failure and Recovery Thereafter. Front Genet, 2020. 11: p. 609262.

3. Ma, H., et al., Aldehyde dehydrogenase 2 (ALDH2) rescues myocardial ischaemia/reperfusion injury: role of autophagy paradox and toxic aldehyde. Eur Heart J, 2011. 32(8): p. 1025-38.

4. Weng, T., et al., Cleavage factor 25 deregulation contributes to pulmonary fibrosis through alternative polyadenylation. J Clin Invest, 2019. 129(5): p. 1984-1999.

5. Masamha, C.P., et al., CFIm25 links alternative polyadenylation to glioblastoma tumour suppression. Nature, 2014. 510(7505): p. 412-6.
