# Supplementary material for: 3′UTR shortening of profibrotic genes and reversibility of fibrosis in patients with end‐stage right ventricular failure
Source: Clin Transl Med. 2022 Sep 9;12(9):e1017. doi: 10.1002/ctm2.1017 (PMC9460478; doi:10.1002/ctm2.1017)
Supplement: Supplementary file 2 — Supplementary material [file CTM2-12-e1017-s001.pptx]

## Slide 1
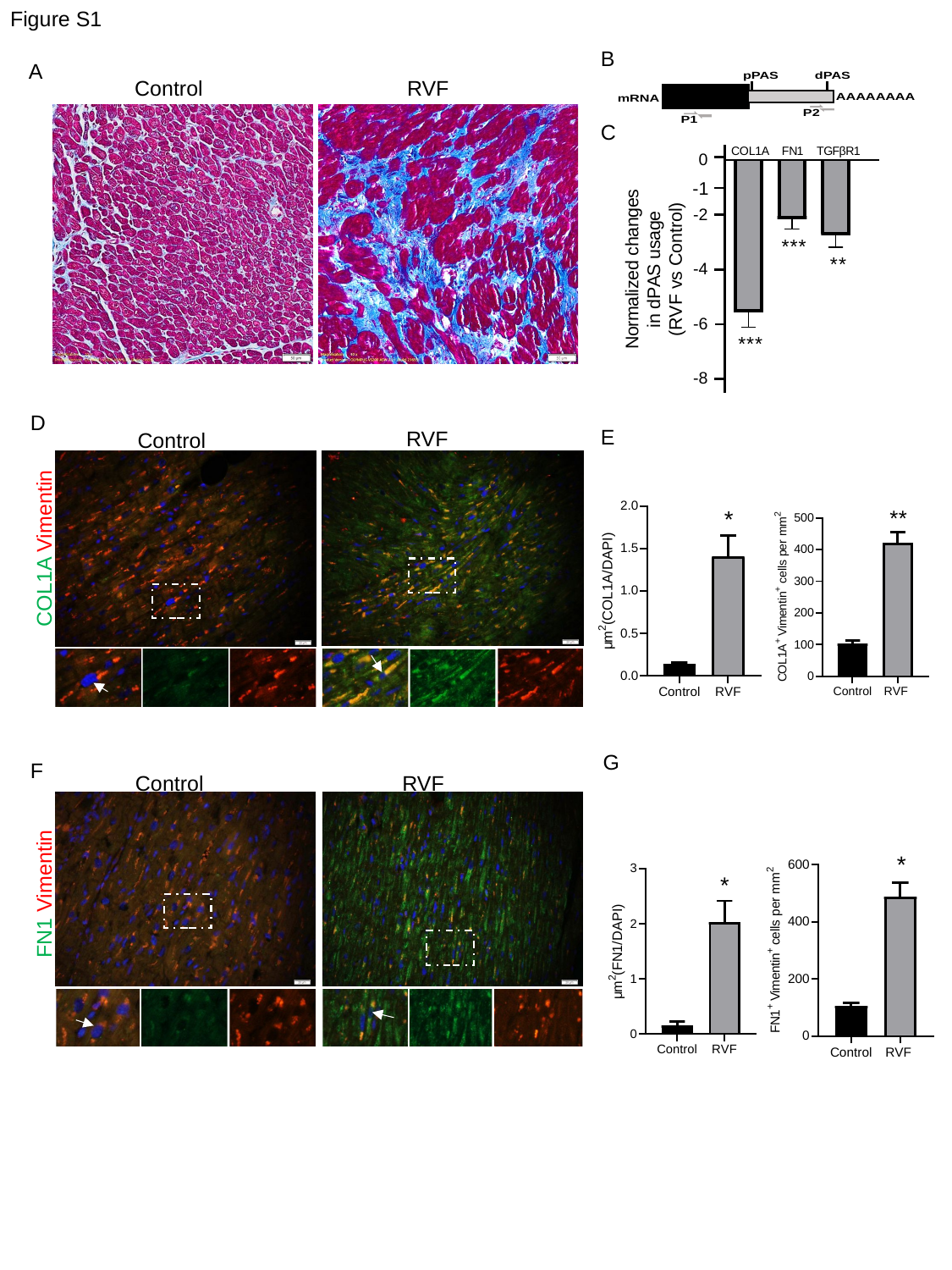

Figure S1
B
A
Control
RVF
C
D
E
RVF
Control
COL1A Vimentin
G
F
Control
RVF
FN1 Vimentin

## Slide 2
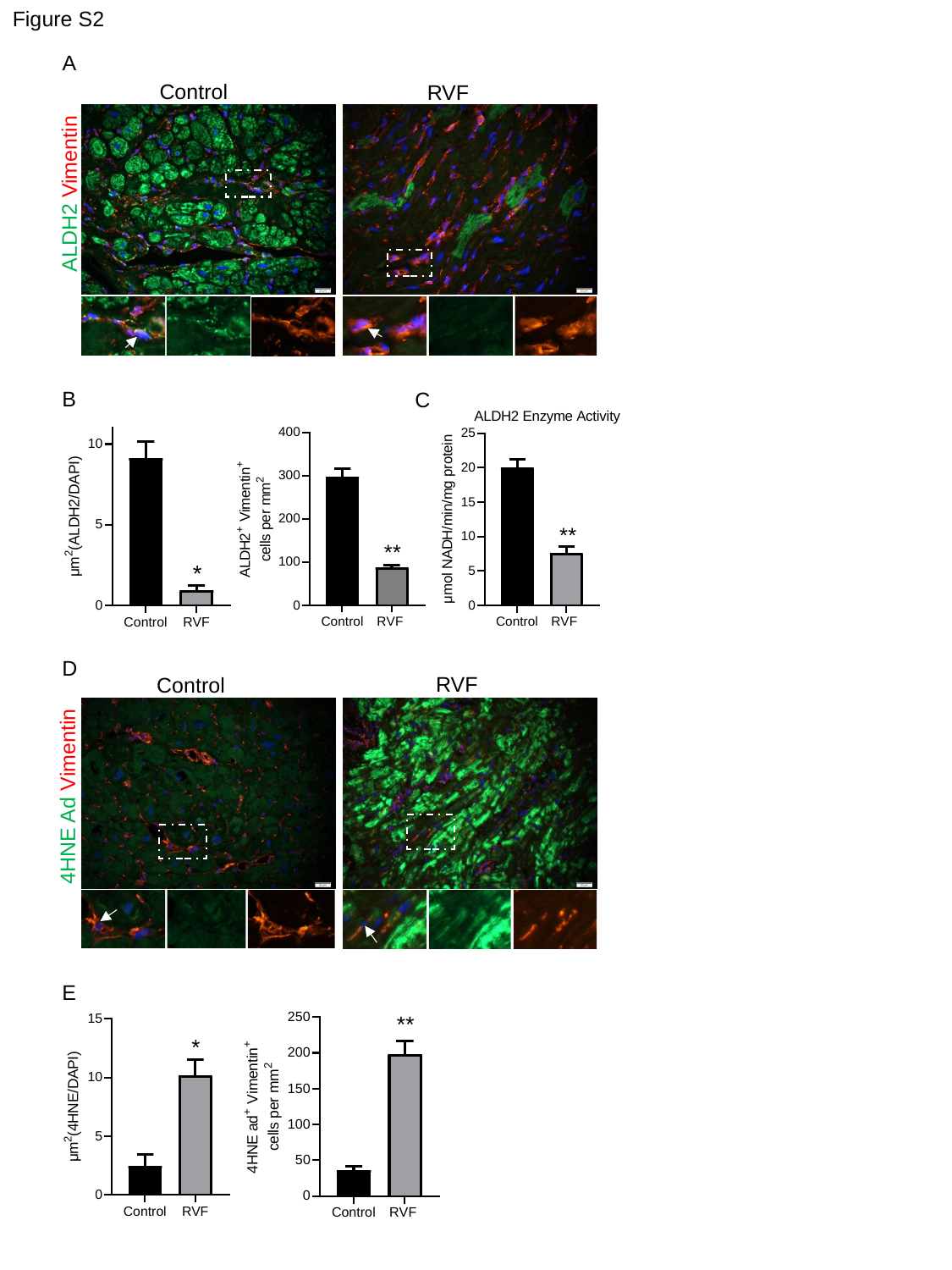

Figure S2
A
Control
RVF
ALDH2 Vimentin
B
C
D
RVF
Control
4HNE Ad Vimentin
E
